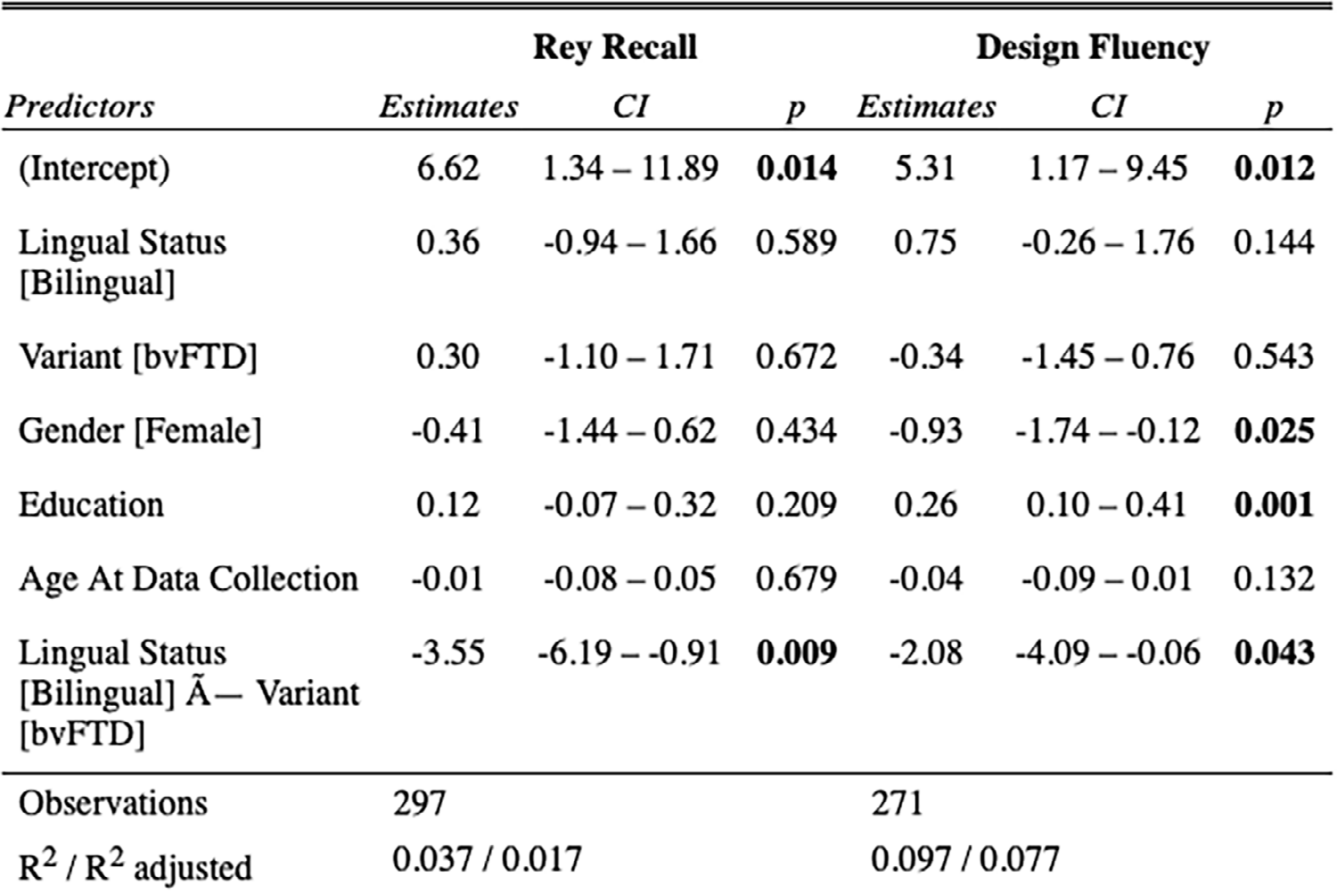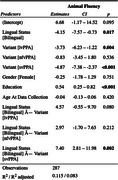# Does Bilingualism Have a Protective Effect on Cognitive Reserve Amongst FTD Variants?

**DOI:** 10.1002/alz70857_098667

**Published:** 2025-12-24

**Authors:** Muskaan Sandhu, Jeanine Sandra Estaban, Janhavi Pillai, Rochelle Reyes, Eugenie Mamuyac, Stephanie M Grasso, Migel Dio, Isabel Elaine Allen, Jessica Deleon

**Affiliations:** ^1^ University of California, San Francisco, San Francisco, CA, USA; ^2^ Memory & Aging Center, Department of Neurology, University of California in San Francisco, San Francisco, CA, USA; ^3^ Memory and Aging Center, UCSF Weill Institute for Neurosciences, University of California, San Francisco, San Francisco, CA, USA; ^4^ The University of Texas at Austin, Austin, TX, USA; ^5^ Memory and Aging Center, UCSF Weill Institute forNeurosciences, University of California, San Francisco, San Francisco, CA, USA, San Francisco, CA, USA

## Abstract

**Background:**

Prior research studies report conflicting evidence regarding the role of bilingualism as a protective factor in cognitive reserve. While it is known that Behavioral Variant Frontotemporal Dementia (bvFTD) and Primary Progressive Aphasia (PPA) disease variants impact distinct underlying neural networks, the effect of being bilingual on these networks is yet to be determined. Here, we investigated whether the impact of language‐related FTD (PPAs) versus non‐language‐related FTD (bvFTD) on cognitive performance varied based on bilingual status.

**Methods:**

This study analyzed a cohort of 316 patients at baseline (224 monolingual and 92 bilingual speakers). These patients were categorized into four disease variants: Logopenic Variant Primary Progressive Aphasia (lvPPA) (*n* = 72), Semantic Variant Primary Progressive Aphasia (svPPA) (*n* = 85), Nonfluent Variant Primary Progressive Aphasia (nfvPPA) (*n* = 79), and Behavioral Variant Frontotemporal Dementia (*n* = 80). Cognitive performance was assessed through 26 neuropsychological tasks across the following domains: general cognition, language, visuospatial, memory, and frontal executive function. We employed multivariate linear regression models with an interaction term between bilingual status and disease variants. Age, education, and gender were included as covariates in the model.

**Results:**

Cross‐sectional results at baseline revealed that bilingual speakers in the bvFTD group scored significantly lower on tasks relating to memory (***Rey Recall***: Bilingual Mean = 4.8 ± 4.2, Monolingual Mean = 7.8 ± 4.6, *p* = 0.009) and visuospatial functioning (***Design Fluency***: Bilingual Mean = 5.1 ± 3.4, Monolingual Mean = 6.3 ± 4.0, *p* = 0.043). On the other hand, svPPA bilingual speakers scored higher on tasks relating to language (***Animal Fluency***: Bilingual Mean = 11.2 ± 8.4, Monolingual Mean = 7.9 ± 4.6, *p* =  0.002). Complete regression outputs are included in Tables 1 and 2.

**Conclusion:**

These findings suggest bilingualism's effects on cognitive performance differ by the FTD variants. This highlights the complexity of bilingualism's role in cognitive reserve and stresses the need to compare within the variants. Future studies should evaluate the trajectory of cognitive performance in bilingual speakers over time compared to their counterparts.